# Circular economy: water quality assessment for irrigation purposes in a constructed-wetland scenario

**DOI:** 10.1038/s41598-025-34161-6

**Published:** 2026-02-02

**Authors:** Patrícia Gomes, Marta Pinheiro, José Martins, Joel Castro, Teresa Valente, Vítor Ribeiro, Marina Mendes

**Affiliations:** 1 Institute of Earth Sciences, Pole of the University of Minho, Universidade do Minho, Campus de Gualtar, Braga, 4710-057 Portugal; 2 PhytoClean (Native Plants), Amares, 4720 Portugal; 3 Landscapes, Heritage and Territory Laboratory (Lab2PT), University of Minho, Campus de Azurém, Guimarães, 4800-058 Portugal; 4 Environmental Division of the Amares Municipality, Largo do Município, Amares, 4720-058 Portugal

**Keywords:** Climate change, Wastewater treatment, Phytoremediation, Reuse and recycling, Non-potable water supply, Ecology, Ecology, Environmental sciences, Hydrology, Water resources

## Abstract

Water is an essential natural resource that sustains life and ecosystems. However, the increasing pressure on freshwater reserves due to climate change, rapid population growth, and industrialization is exacerbating the issue of water scarcity. In this context, wastewater reuse has emerged as a vital strategy to address water shortage. Also, it supports the United Nations Sustainable Development Goals and aligns with the principles of the circular economy. In this context, phytoremediation appears to be a viable solution that employs plant species to purify water, thereby contributing to water reuse. So, this study focuses on evaluating the feasibility of using treated wastewater from a constructed wetland for irrigation purposes. The investigation involved establishing a comprehensive monitoring plan, including sampling and analytical processes, followed by in situ and laboratory analyses of the collected water samples. The results indicate that the treated wastewater met the quality standards set by National and European legislation for irrigation purposes. Some parameters, such as chemical oxygen demand, total suspended solids, and turbidity, demonstrate high removal efficiencies, with maximum removal efficiencies exceeding 97%. The anions and potentially toxic elements showed very low values, being within the standards for water reuse for irrigation, except ammonium (NH_4_^+^), which did not comply with the standards in any of the campaigns. The SAR, with a value of less than 2, was below the maximum recommended value (MRV) of 8. Overall, the findings support the use of treated wastewater from constructed wetlands for irrigation, which offers an effective solution for water reuse and contributes to environmental sustainability.

## Introduction

Water is a crucial natural resource that sustains life and the environment. The two primary categories of natural water resources, surface water and groundwater, play indispensable roles in meeting various human needs^1^. However, the escalating demands resulting from climate change, rapid population growth, overexploitation of natural resources, and accelerated industrialization have imposed immense pressure on freshwater reserves^2^. About 90% of the worldwide wastewater drains into surface water bodies in untreated conditions^3^. At these locations, water bodies, including rivers, lakes, and coastal waters, have typically been severely impacted by the release of contaminants into them^4^. These untreated contaminants seriously impair surface water quality and threaten the security of aquatic ecosystems^5^. Consequently, effluents inevitably cause an increase in organic and inorganic waste, instigating water contamination^6^.

The quality of water resources is essential to promoting economic development and conserving ecosystems, thus fulfilling sustainability goals^7^. The world can depend on some potential water resource options in the future to promote sustainable development. In Europe, roughly 81% of freshwater resources are used for agricultural, drinking, and industrial purposes, while less than 3% of urban wastewater is currently reused^8^. The same authors report that approximately 1 billion cubic meters of treated urban wastewater are reused annually in the EU, representing only around 2.4% of treated urban effluents and less than 0.5% of total annual freshwater withdrawals. Furthermore, according to the European Environment Agency, Portugal ranked 5th in the EU Water Exploitation Index in 2019, experiencing significant seasonal water scarcity (seasonal WEI + > 40%)^9^. Therefore, wastewater reuse and resource recovery, in line with the circular economy concept, are considered promising approaches in combating global water scarcity and making significant contributions to sustainable development^10^. Compared to other types, municipal wastewater is easier to reuse because it originates from domestic and non-industrial sources and contains less pollution^11^. Therefore, there is a great need for new technologies. Innovative and sustainable technologies have been developed to reduce water consumption and mitigate environmental impacts^12^. According to the European Commission, treating wastewater at urban wastewater treatment plants is a viable option to appease the increasing demand for water resources^13^. Reusing treated wastewater for non-potable purposes can also help augment the water supply in water-scarce areas. It can tackle water stress and improve the groundwater table through recharge after suitable quality standards have been achieved^14^.

In this context, water scarcity is currently considered a major challenge for Europe, and this scenario is being rapidly exacerbated by climate change. To address this issue, the European Commission proposed in June 2018 a regulation^15^ on minimum requirements for water reuse in irrigation and aquifer recharge^16^. Furthermore, Portugal approved a new policy in August 2019^17^ that portrays water production for reuse from several sources (urban, domestic, industrial) for multiple non-potable purposes such as agriculture irrigation, urban uses, or even ecosystem support. The new water reuse policy’s goals include integrating water reuse at the European level, developing various non-potable applications, and establishing a flexible management approach that prioritizes health and environmental safety^18^. To support these goals, the Portuguese Environment Agency developed a guideline providing guidance on permitting procedures and technical support for health and environmental risk assessments^19^.

Given the increasing importance of water reuse, various treatment methods are being explored to ensure the water meets the required quality standards. Among these methods, phytoremediation is a valuable way for the cleanup of extensively polluted water bodies, effectively targeting the removal of both organic and inorganic pollutants^20^. While phytoremediation broadly refers to the use of plants to remove, stabilize, or degrade contaminants in soil, sediment, or water^21^, constructed wetlands (CWs) are engineered systems specifically designed for treating contaminated water. So, constructed wetlands provide a promising nature-based solution (NbS) to these challenges, utilizing the natural processes of wetland plants, substrates, and associated microorganisms to improve water quality^22^. This approach boasts low energy consumption, requires minimal investment, and achieves effective treatment outcomes. However, the success of constructed wetland treatments depends on aspects of their construction, such as the type of vegetation, flow, and surface level of the wastewater^22,23^.

Thus, the work presented is part of a broader investigation into the feasibility of treating wastewaters through CW. Specifically, it addresses the research gap concerning the effectiveness of constructed wetlands for removing contaminants while producing water suitable for reuse in irrigation. The objectives are (i) to evaluate the treatment performance of the wetland system, (ii) to assess the quality of the treated effluent in relation to reuse standards, and (iii) to examine its potential impacts on soils. By doing so, the study offers practical insights for implementing sustainable water reuse strategies in alignment with national and European regulatory frameworks, thereby contributing to the achievement of the United Nations Sustainable Development Goals^7^ on water management and environmental protection.

## Study area

**Fig. 1:**
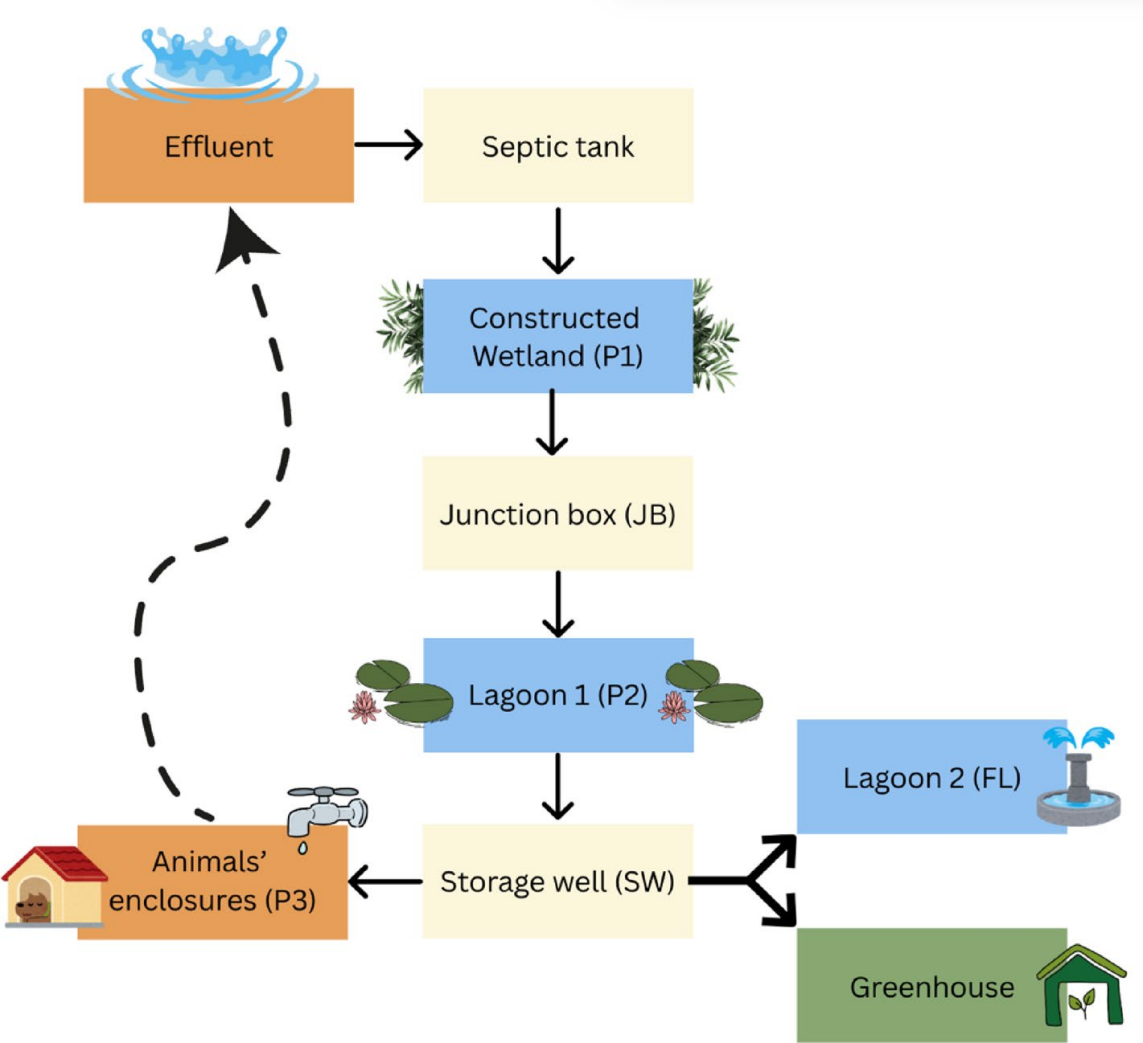
Schematic layout of the eco-friendly dog shelter, with emphasis on water circulation. P1 – Constructed wetland (CW); P2 – Lagoon 1; P3 – Water outlet. (**a**) Fountain lagoon (FL); (**b**) Bottom of the CW (BCW); (**c**) Junction box (JB); (**d**) Storage well (SW).

The study area was chosen because it hosts a fully operational constructed wetland (CW) that treats wastewater on-site. This makes it a practical and representative case for evaluating the efficiency of CW-based water treatment and the potential for water reuse under the specific environmental and climatic conditions of northern Portugal. The CW is located in the municipality of Amares (Braga district), at a dog shelter recognized as the first eco-friendly dog shelter in northern Portugal and considered a national reference project. Beyond promoting animal adoption, the shelter incorporates circular economy practices, including water reuse, biomass recycling through composting, and the use of sustainable building materials. The CW, opened in 2021, is operated and maintained by the Amares Municipality to ensure continuous treatment performance. Figure 1 presents a schematic layout of the shelter and the water circulation system.

The treatment process begins when the effluent enters a septic tank for primary settling, followed by its flow into the CW (P1), where specific plant species contribute to further purification before the water reaches the first lagoon (P2)^23^. This lagoon enhances phytoremediation and promotes the removal of contaminants through exposure to ultraviolet radiation. The treated water is then collected in a storage well (SW), from which it can be reused via a dedicated outlet (P3). Reclaimed water is employed for cleaning animal enclosures, supplying the sanitary facilities, and irrigating trees in the greenhouse. A second lagoon (FL), with an ornamental function, receives part of the treated water in addition to collecting rainwater (Fig. 1).

**Fig. 2:**
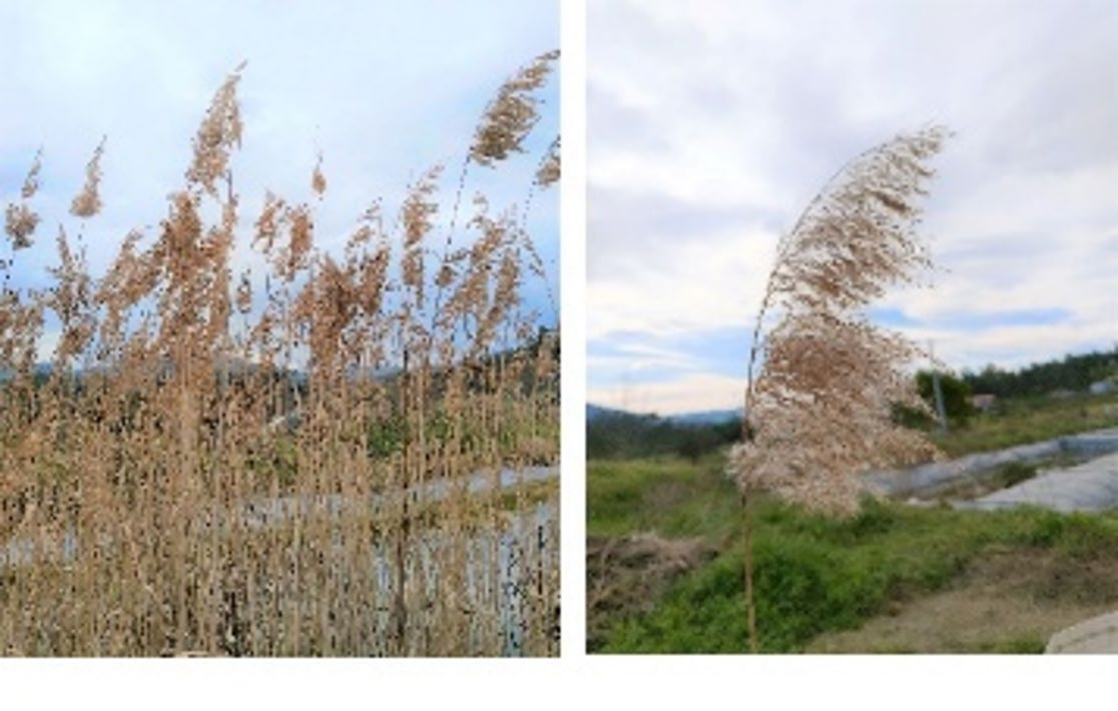
*Phragmites australis* in its natural habitat (photographs taken by the authors).

At the study site, three functional groups of macrophytes were introduced: emergent species (rooted in sediment with leaves extending above the water surface), floating species (leaves on or just below the water surface), and submerged species (entirely underwater)^24^. In the CW itself, only one macrophyte species is present: *Phragmites australis* (Fig. 2). This is the most widely used species in constructed wetlands across Europe^25^, owing to its high adaptability to environmental variability, rapid growth (up to 6 m in height), high biomass production, and extensive rhizome system^21,26^. Its phenology includes a flowering period from July to September, followed by senescence.

Climatic data from the past 30 years, as well as for 2021/2022, were analyzed for the northern region of Portugal. Mean annual temperature ranged from 11 to 12 °C, with July and August being the warmest months. Annual precipitation averaged ~ 2700 mm, with most rainfall occurring between December and March^27^. These climatic conditions are directly relevant to the study site, since periods of reduced precipitation coincide with lower water availability and higher influent concentrations. For this reason, field campaigns were scheduled during the dry season, ensuring that the system was assessed under limited water availability and, consequently, the most demanding local climatic conditions.

## Materials and methods

### Sampling and analytical procedures

#### Sampling design and frequency

Five field campaigns were conducted between March and July 2022 to characterize the performance of the constructed wetland (CW). Sampling focused on three main points along the treatment line (Fig. 1):

- **P1** – influent, collected at the outlet of the septic tank, where raw wastewater enters the CW;
- **P2** – Lagoon 1, after phytoremediation in the CW;
- **P3** – final water outlet, where treated water is reused in the shelter.

To complement system characterization, additional monitoring points were included: fountain lagoon (FL), bottom of the CW (BCW), junction box (JB), and storage well (SW). Together, these locations provided an integrated view of treatment processes and water circulation within the shelter.

#### Field procedures

At each campaign, in situ measurements were taken using a HACH HQ30d flexi multiparameter: pH, electrical conductivity (EC), total dissolved solids (TDS), water temperature (T), dissolved oxygen (DO), salinity, and redox potential (Eh). In addition, color (by direct observation) and odor were qualitatively assessed.

Water samples were collected at P1, P2, and P3 in 1-L high-density polyethylene bottles (Kartel). Two additional 50-mL polyethylene bottles per site were prepared after filtration through 0.45-µm Millipore membranes: one for anion analysis, and another acidified with HNO_3_ to pH ~ 2 for cation determination (applied in the 1st and 4th campaigns only). All containers were pre-labeled (site/date) according to ASTM 5245^28^, stored under refrigeration, protected from sunlight, and immediately transported to the laboratory.

#### Laboratory analyses

- Anions (F⁻, Cl⁻, NO_2_⁻, Br⁻, PO_4_^3^⁻, NH_4_⁺, NO_3_⁻, SO_4_^2^⁻): determined by ion chromatography with suppressed conductivity detection (761 Compact IC Metrohm, Herisau, Switzerland). Detection limits: 0.03 mg/L (PO_4_^3^⁻), 0.01 mg/L (others). Measurement precision: <5% RSD.
- Alkalinity: volumetric titration (Standard Methods 2320 B)^29^.
- Chemical oxygen demand (COD): photometric determination (Hanna Instruments) using medium-range reagents (0–1500 mg/L). Digestion was carried out for two hours at 150 °C in an HI839800 reactor, followed by photometric measurement (HI83399).
- Turbidity: turbidimetry (TB1000), analyzed in triplicate (Standard 4500-SO_4_^2^⁻ E).
- Total suspended solids (TSS): gravimetric determination after filtration and drying of glass microfiber filters (Standard Methods 2540 D).
- Potentially toxic elements (PTEs: Li, Be, Al, V, Cr, Mn, Fe, Co, Ni, Cu, Zn, As, Se, Mo, Cd, Sn, Ba, Pb): analyzed by ICP-MS at Actlabs (Ontario, Canada). Accuracy verified using certified reference materials; precision < 5% RSD. Reagents were of analytical grade or Suprapur (Merck, Darmstadt, Germany). Milli-Q water was used in all experiments, and standards were of Merck AA Certificate grade.

### Graphical methods

The sodium absorption ratio (SAR), a critical indicator for irrigation water, was calculated as:

$$\:SAR=\frac{r{Na}^{+}}{\sqrt{\frac{r\left({Ca}^{+2}+{Mg}^{+2}\right)}{2}}}$$

where concentrations are expressed in meq/L. For the graphical analysis, the AquaChem 5.1 software was used.

## Results and discussion

### Hydrochemical properties—legal compliance

To assess irrigation water quality, Portuguese national standards were applied. These standards, derived from European Union regulations, ensure that the assessment adheres to the most conservative criteria. Also, it is important to notice that, in this study, the evaluation of irrigation water quality was conducted in accordance with Decree-Law 236/98^30^, which sets criteria and quality standards aimed at protecting public health, the quality of surface and groundwater, crops susceptible to contamination, and soils whose agricultural suitability could be affected by the use of poor-quality irrigation water. Given that this work also considered the potential adverse effects on soils and aquifers, Decree-Law 119/2019^17^, which regulates the reuse of treated wastewater, was additionally referenced. Although different categories of water reuse exist, the focus of this study was on the irrigation of forest species present in the kennel area. So, to assess the possibility of using the treated influent for irrigation and promoting the concept of a circular economy, Table 1 displays the results of the parameters analysed in situ. The results indicate that site FL—1st campaign—has the highest pH value (9.42), while sampling point P1 in June had the lowest value (6.81). The results show that throughout the campaigns, these points tended to exhibit the highest and lowest pH values, respectively.

**Fig. 3:**
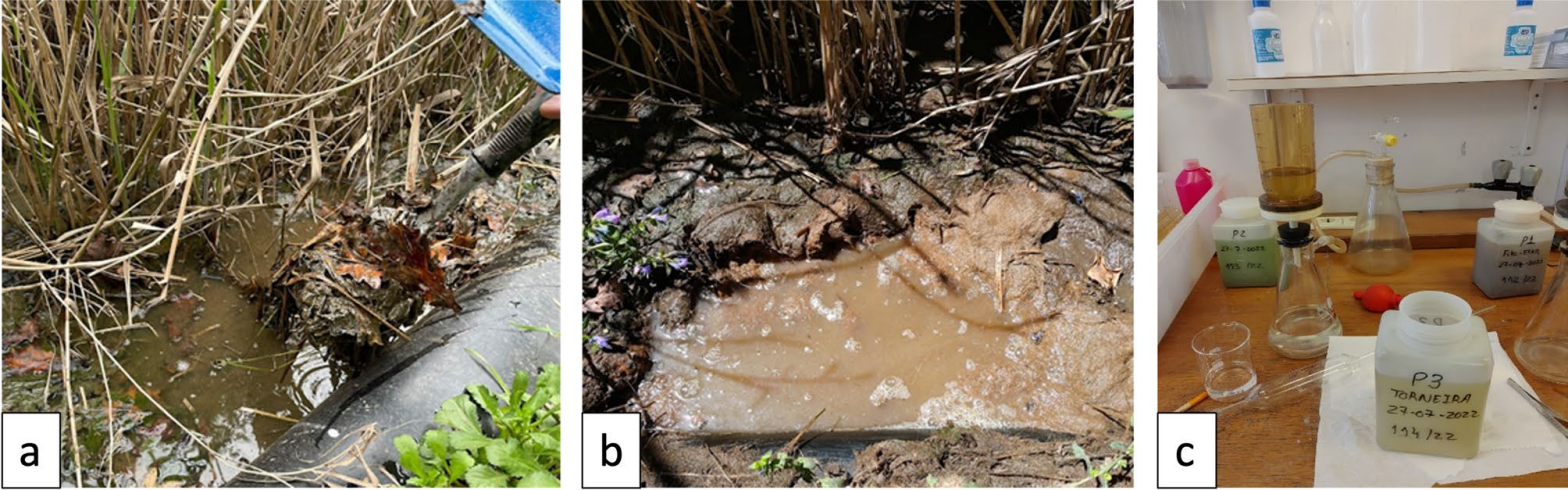
Organic load at the beginning of the constructed wetland (P1): (**a**) Organic matter layer—1st campaign; (**b**) Animal waste—4th and 5th campaigns; Observation of water color in the bottles – P1: brown; P2: greenish; P3: greenish (photographs taken by the authors).

Regarding EC, it exhibited some variability, with the highest value recorded at 1142 µS/cm in June at site P1 and the lowest at 333.9 µS/cm in May at site C1. The EC of 500 µS/cm in the third campaign (P1) was somewhat inconsistent when compared to the other readings at the same location. Given that this point is a primary treatment site—the beginning of the CW—high organic load was frequently observed during the sampling campaign (Fig. 3). This lower value may be associated with the heavy rainfall that occurred on the days preceding the campaign, likely causing a dilution effect as water washed away salts through runoff inflows^31^. This suggests that the decrease in electrical conductivity follows an increase in water level, reflecting a rise in flow rate that enhances sediment transport capacity^32^. The recorded temperatures appeared consistent with the local climate^33^.

The lowest temperature recorded, 13.2 °C, occurred during the first campaign, while the highest was 28.8 °C in the last campaign.

The oxygen parameter varied significantly among the analyzed sites. The highest concentration, 24.71 mg/L (FL), was recorded in March, while the lowest, 0.14 mg/L, was in April (P1). Generally, the FL and P2 sites consistently showed the highest O_2_ values, likely because both sites are lagoons that benefit from aeration due to their exposure to the atmosphere, thereby increasing oxygen levels^34^. In contrast, the lowest oxygen concentrations are found in confined areas, such as the storage well (SW) and sampling point P3, where treated water exits—water outlet. Among these sites, P1 (CW) also stands out, with the low oxygen levels attributed to biological processes rather than limited oxygen contact. According to^35^, constructed wetlands are systems that rely on natural processes involving vegetation, soil, and microbial communities to treat wastewater. These systems engage in various biological activities that can lower oxygen levels. For example, the continuous saturation of the filtration beds can promote anoxic/anaerobic conditions, which in turn contribute to lower oxygen levels^35^. Additionally, the type of plants used in CW systems plays a critical role, as aquatic macrophytes influence oxygen dynamics by releasing oxygen into the rhizosphere through their roots, affecting the biogeochemical cycles of the surrounding sediments by altering the redox state^36^.

The TDS values were similar throughout the sampling points except for site P1 in the third campaign, which was slightly lower, following the trend of the EC value. The junction box (JB) sampling point surpassed the MRV of 640 mg/L in all campaigns. On the other hand, the FL site, which consistently showed lower results than the other samples, had the highest value of 157 mg/L in May.

Salinity remained stable throughout the campaigns, with a minimum value of 0.24% in April and a maximum of 0.59% in March. Since the effluents under treatment were always the same type, no significant variation in this parameter was observed.

Color observation revealed consistent patterns across the sampling points: P1 consistently exhibited a brownish color, P2 displayed a more greenish color, and P3 varied between greenish and clear, with the clear color occurring only in June (Fig. 3c). This observation may suggest a higher efficiency of nutrient removal, potentially reducing the microalgae that typically color the water, as noted by^37^. Wetlands have been shown to effectively reduce algal growth and retain nutrients, both of which are key factors influencing watercolor. The odor parameter at site P3 could not be analyzed during the first campaign due to the strong smell emanating from the nearby animal enclosures. Site P1 consistently exhibited the strongest odor, likely due to the elevated water levels in the CW being above the substrate level (Fig. 3a, b). The water level may also be related to the fact that March was a rainy month, suggesting a potential increase in the flow of the CW, which could create difficulties in its operation^33^. According to^38^, the water level should always be below the substrate, thus reducing the smell and possible accumulation of mosquitoes on the surface of the water. The heat is another significant factor, as it intensifies the odor of the effluent at the CW entrance. These are some of the reasons for the necessary maintenance of CWs, which may not affect their viability but can lead to discomfort and embarrassment during operation^38^.

The redox potential (Eh) is a key indicator of oxidation-reduction conditions in constructed wetlands, where it can range from strongly reduced conditions (− 500 mV) to moderately oxidized environments (+ 500 mV), reflecting the influence of plant species and the environmental conditions^39,40^. The most reduced value recorded was − 227 mV in April at site P1, while the most oxidized value was + 143 mV in July at site P2. Data analysis reveals that lower Eh values are correlated with a higher COD, increased internal oxygen demands, and oxygen transfer efficiency^41^. The Eh parameter is significantly influenced by the bed’s filling characteristics, including porosity, which facilitates oxygen exchange between the substrate and the macrophytes, ultimately impacting microbial activity and nutrient removal efficiency^42^. Most of the sites analyzed exhibited a reducing environment. The SW sampling point from the second campaign was not analyzed due to the water level being too low for measurement.

The analyses indicate that all these sites need to be addressed more efficiently. Site P3 has the highest remediation efficiency, enabling the potential reuse of treated water.

To deepen the research carried out, Table 2 presents the results of the parameters analyzed in the laboratory for the sampling points (P1, P2, P3) of the five campaigns conducted between March and July 2022.

**Table 1:** Results of the in situ analyses of parameters from the sampling points P1, P2, P3, and the monitoring points: fountain lake (FL), bottom of the CW (BCW), junction box (JB), and storage well (SW).

Campaign (2022)	Sampling points	pH	EC (µS/cm)	T (°C)	O_2_ (mg/L)	TDS (mg/L)	Salinity (%)	Color	Odor	E_h_ (mv)
March	P1	7.03	1115	14.1	0.83	547	0.59	Brownish	Strong	− 210
	P2	8.34	953.9	16.5	18.3	467.9	0.51	Greenish	None	− 7
	P3	7.77	952.4	13.2	2.5	467.2	0.5	Greenish	–	− 23
	FL	9.42	333.9	17.1	24.71	164.1	0.208	Greenish	Slight	48
	BCW	7.42	904	13.4	0.46	443.5	0.485	Brownish	Slight	− 198
	JB	6.97	1360	11.4	0.25	668.1	0.71	Clear	None	− 228
	SW	8.35	961.3	15.7	17.13	471.6	0.517	Greenish	None	13
April	P1	7.26	857	15.5	0.14	420	0.42	Brown	Slight	− 227
	P2	7.59	890	23.7	0.75	437	0.44	Greenish	None	− 32
	P3	7.64	899	22.5	0.28	441	0.44	Greenish	None	− 176
	FL	9.37	377	21.4	15.22	180.5	0.18	Greenish	None	− 12
	BCW	7.08	758	20.8	4.49	353	0.35	Greenish	None	− 88
	JB	6.73	1595	14	0.41	759	0.76	Clear	None	− 197
	SW	7.96	915	16.5	0.4	449	0.45	Greenish	None	–
May	P1	7.05	500	20.7	4.36	241	0.24	Brownish	Slight	83
	P2	7.79	1073	21.6	13.66	528	0.53	Greenish	None	137
	P3	7.38	1135	22.5	0.62	533	0.53	Brownish	Slight	− 74
	FL	9.23	343	23.3	13.15	157	0.16	Turbid	None	105
	BCW	7.45	915	19.5	0.9	448	0.45	Turbid	None	− 128
	JB	6.8	1351	17.4	0.34	669	0.66	Clear	Slight	− 111
	SW	8.11	937	20.7	0.3	439	0.44	Clear	None	114
June	P1	6.81	1142	20.5	0.29	557	0.56	Brown	Strong	− 68
	P2	7.99	1109	25.1	6.47	537	0.54	Brownish	None	75
	P3	7.53	994	21.3	0.17	484	0.48	Clear	Slight	− 53
	FL	7.79	490	25.8	4.93	234	0.23	Greenish	None	109
	BCW	7.94	1086	22.4	1.24	529	0.53	Brownish	Slight	− 139
	JB	6.82	1219	18.6	0.22	664	0.67	Brownish	Slight	− 131
	SW	8.01	935	18.4	0.15	454	0.46	Clear	None	− 60
July	P1	7.1	1086	28.5	0.3	529	0.53	Brown	Strong	− 96
	P2	7.53	952	28.8	21.41	462	0.46	Greenish	None	143
	P3	7.82	1047	24.7	0.65	509	0.51	Greenish	Slight	− 14
	FL	9.13	441	29.8	9.7	211.4	0.21	Greenish	None	112
	BCW	7.31	1249	26.3	2.44	611	0.61	Turbid	None	− 32
	JB	6.9	1583	23.3	0.34	781	0.79	Clear	None	− 130
	SW	8.03	823	25.7	1.05	398	0.4	Turbid	None	− 37
	Average	7.5 (15)	980 (15)	21.2 (15)	4.7 (15)	477 (15)	0.49 (15)	–	–	36.1 (15)
	MRV	6.5–8.4	1000	–	–	640	–	–	–	–
	MPV	4.5–9.0	–	–	–	–	–	–	–	–

The maximum recommended value (MRV) and maximum permissible value (MPV) correspond to DL No. 236/98, which regulates water quality for irrigation. The averages presented correspond to the sampling points P1, P2, and P3.

**Table 2:** Results of the parameters analyzed in the laboratory of sampling points P1, P2, and P3.

Parameters	Campaigns															MRV	MPV	MPV
	March/2022			April/2022			May/2022			June/2022			Jully/2022					
	P1	P2	P3	P1	P2	P3	P1	P2	P3	P1	P2	P3	P1	P2	P3			
COD	369	189	66	273	124	50	426	154	40	1235	228	85	1500*	228	44	–	–	150
Alkalinity	546.25	458.75	423.75	484.5	411.25	395	407.5	520	480	520	507.5	440	540	585	485	–	–	–
TSS	2748	1112	327	410	28	1.5	168	190	7.5	1470	408	7.3	392	456	38.5	60	35 *	–
Turbidity	204.7	60.2	4.03	60.5	8.79	5.58	83.4	86.05	8.19	858	92.5	9.13	738.3	91	26.4	–	–	–
F^−^	0.22	0.29	0.37	0.24	0.4	0.39	0.39	0.33	0.09	0.46	0.24	0.16	15.5	0.17	0.15	1 (2)*	15	–
Cl^−^	29.29	29.75	30.87	30.16	25.64	26.18	23.77	25.24	25.63	30.45	36.09	29.69	25.56	29.67	28.16	70	–	–
NO_2_^−^	0.62	0.42	0.36	0.29	0.24	0.31	0.31	0.35	0.44	0.08	0.44	0.45	0.28	0.23	0.39	–	–	–
Br^−^	1.41	1.81	1.52	1.99	1.31	1.46	1.89	1.45	1.5	1.94	1.83	1.59	1.98	1.22	1.72	–	–	–
NO_3_^−^	0.23	0.27	0.09	0.01	0.2	0.03	0.02	0.03	0.14	< 0.01	0.06	0.03	0.04	0.38	0.03	50	–	50
PO_4_^3−^	34.45	65.44	41.38	61.85	38.91	50.14	44.37	41.35	42.17	96.34	96.7	84.35	87.52	48.93	67.28	–	–	–
SO_4_^2−^	1.09	1.28	5.14	1.25	1.08	3.4	10.57	4.09	3.13	2.97	7.59	3.91	8.71	6.59	3.13	575	–	2000
NH_4_^+^	100.5	73.7	83.1	108.6	88.5	96.4	92.8	109.4	87.8	137.5	65.3	157.4	101.5	82.1	100,3	–	–	10

The maximum recommended value (MRV) and the maximum permissible value (MPV) correspond to DL No. 236/98 regarding the quality of water intended for irrigation and the emission limit value (MPV) regarding wastewater discharge. All results are expressed in mg/L, except turbidity, which is presented in NTU, and alkalinity, which was measured in mg/L of CaCO_3_. (Chemical Oxygen Demand – COD; Total Suspended Solids – TSS).

*Value corresponding to DL No. 119/2019, Class D.

Regarding the COD parameter, the highest value recorded was 1500 mg/L at site P1 during the last campaign, while the lowest was 40 mg/L at site P3 in May, with an average COD of 251 mg/L. Generally, these sites exhibited the highest and lowest COD values, as expected. As previously mentioned, site P1 indicates the beginning of the CW, marking the start of the treatment process. Site P3 is the endpoint, or water outlet, where the treated water is used. According to^30^, the maximum permitted discharge value is 150 mg/L. Therefore, all campaigns remained within the emission limit value. It is important to note that during the last campaign, animal waste was found in the pipe leading into the CW, along with significant plant biomass (which could potentially obstruct the water inlet) and decreased water circulation. These factors can result in an accumulation of organic matter, which helps clarify the elevated COD levels. According to several authors^43,44^, the COD values increase with a higher amount of organic load inserted into the environment. This may be attributed to two reasons: reduced rainfall (in the context of extreme climate change) and inadequate maintenance of the CW itself, specifically the seasonal cutting of vegetation at a time when it would be necessary due to its rapid growth. This is particularly important, as^45^ observed that the removal efficiency decreases with increasing COD concentrations. According to^23^, maintenance activities should include the periodic removal of accumulated debris, inspection of inlet and outlet structures, and monitoring and cutting of vegetation. Ensuring regular water circulation and avoiding long-term storage can significantly enhance the efficiency of these systems.

The analysis of TSS reveals suggestive variability among sampling points. As expected, sampling point P1, located at the beginning of the CW, exhibits particularly high TSS values, reaching a maximum of 2748 mg/L. At site P2, there is an immediate decline in TSS concentrations. This decrease may be attributed to the more effective removal of TSS occurring early in the inflow into the CW. In constructed wetlands, the presence of high organic matter at the inlet promotes microbial activity, enhancing the removal efficiency^46^. This highlights that microbial communities are crucial for understanding performance patterns and optimizing treatment strategies in CWs^47^. Additionally, the retention time and the type of substrate play significant roles in the TSS removal process^48^. At site P3—water outlet—the March campaign showed a slightly higher concentration of 327 mg/L. However, during the campaigns, lower values were recorded, consistent with the requirements of National legislation^30^, which establishes an MRV for TSS at 60 mg/L. Under Decree-Law No. 119/2019^17^, specifically regulated for reuse purposes, TSS values must be ≤ 35 mg/L. Therefore, the results from March and July demonstrate non-compliance with this legislation. Overall, the March campaign recorded the highest values, while the April campaign recorded the lowest.

Concerning turbidity, the values shown represent the averages from three replicates conducted at each location, measured in NTU. Table 2 shows that sampling point P1 exhibits exceptionally high turbidity levels in the water. These values are expected, given that the site receiving the septic tank influent has no previous treatment and successively accumulates organic and inorganic matter. This site had a maximum turbidity of 858 NTU in the June campaign. Sampling point P2 already shows lower values, with a maximum of 92.5 NTU. Meanwhile, site P3, which represents the endpoint of the effluent purification process—water outlet—displayed the lowest turbidity levels, recording a maximum of 26.4 NTU and a minimum of 4.03 NTU in March and July, respectively. This demonstrates the impact that climatic conditions have on the parameters analyzed, and the crucial need to study the different hydrological seasons in this type of system.

According to^2^, most CWs can provide reliable levels of treatment, evidenced by TSS, for example. However, they are often less effective at removing certain nutrients. According to the same authors, this setback could potentially be reversed with improved oxygenation. Regarding the ammonium (NH4+) values, these are quite high. The CW under study appears incapable of achieving sufficient nitrification to reduce the ammonium concentration to the 10 mg/L limit established by the Decree-Law No. 236/98^30^. In certain circumstances, the concentration of ammonium upon entering the CW is lower than the concentration of ammonium when the effluent exits the CW bed, as observed during the June campaign. In other words, instead of a reduction as expected, there is an increase in ammonium production. This may result from the anaerobic decomposition of organic nitrogen trapped in the beds. Due to the anaerobic conditions, oxygen is insufficient to facilitate ammonium removal, as the NH4 + removal rate is higher in aerated areas where nitrification tends to occur^49^. One observation at the study site was the low use of treated water at site P3, which leads to increased stagnation in the CW bed, ultimately detrimental to the treatment. An effective solution could involve improving water circulation in the treatment circuit. According to^50^, most CW systems with horizontal flow can reliably treat TSS. However, they are often less effective for nitrogen removal unless they provide a longer hydraulic retention time and sufficient oxygenation^43^.

**Fig. 4:**
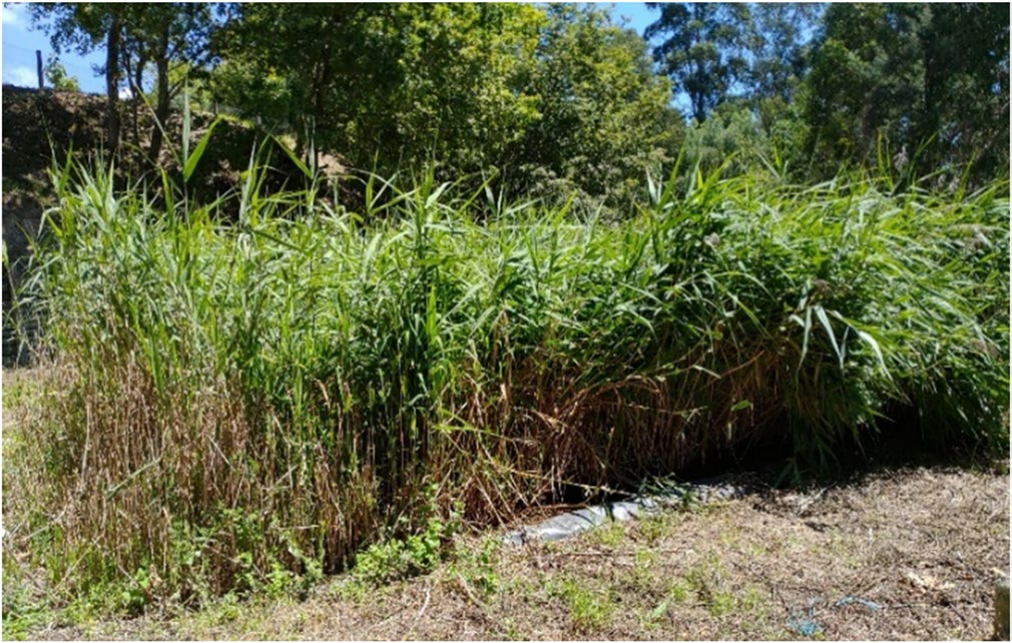
Phragmites australis present in the constructed wetland (CW) during the June campaign (photographs taken by the authors).

Accordingly, the oxidized forms of nitrogen (NO_2_^−^ and NO_3_^−^) exhibit very low values at all sampled points. These parameters are thus significantly below the ELV of 50 mg/L. The removal of the different forms of nitrogen also depends on healthy plant growth, as nitrification and plant absorption are the primary mechanisms for removing this element^51^. This was observed, and the plants showed good vegetative growth (Fig. 4). As this type of macrophyte bed is mostly anaerobic, the formation of nitrites and nitrates occurs through the oxidation of ammonium (nitrification) in areas where dissolved oxygen concentrations are above 1 mg/L^52,53^, well above the concentrations detected.

However, as mentioned earlier, thinning the aerial parts is crucial for improving water circulation and enhancing the effectiveness of the CW. Maintenance, regarding the removal of stagnant organic matter from the CW, such as decaying leaves, would also be an essential aspect.

Regarding sulfate, it does not exceed the MPV of 575 mg/L set by legislation and is significantly below the VLE. It varies between sampling points but remains within the required range limits.

Table 3 presents the results of PTEs analyzed during the first and fourth campaigns. These elements were selected because they are included in Decree-Law No. 119/2019, Annex I, concerning water quality standards for reuse in irrigation, and in Decree-Law No. 236/98, Annex XVI, which addresses water quality for irrigation purposes. Furthermore, the table highlights the maximum recommended and permissible values as specified by the Ministry of the Environment^30^.

**Table 3:** Results of the potentially toxic elements (PTEs) analyzed in the March and June campaigns.

Parameters	P1 Mar	P2 Mar	Campaigns				MRV	MPV	QS
			P3 Mar	P1 Jun	P2 Jun	P3 Jun			
Li	0.033	0.022	0.023	0.048	0.035	0.029	2.5	5.8	2.5
Be	< 0.0002	< 0.0002	< 0.0002	< 0.0001	< 0.0001	< 0.0001	0.5	1	0.1
Al	0.024	0.017	0.016	0.056	0.024	0.049	5	20	5
V	< 0.0002	0.0003	0.0002	0.0002	0.0004	0.0004	0.1	1	0.1
Cr	< 0.001	< 0.001	< 0.001	< 0.0005	< 0.0005	< 0.0005	0.1	20	
Mn	0.0607	0.14	0.0884	0.043	0.0327	0.0589	0.2	10	0.2
Fe	0.23	0.35	0.05	0.19	0.15	0.23	5		2
Co	0.000201	0.00027	0.000185	0.000469	0.000412	0.000724	0.05	10	0.05
Ni	0.0013	0.0009	< 0.0006	0.002	0.0011	0.0017	0.5	2	
Cu	0.001	0.0013	< 0.0004	0.0034	0.0015	0.0246	0.2	5	
Zn	0.0264	0.0154	0.0114	0.0243	0.0171	0.0264	2	10	
As	0.00397	0.0131	0.0151	0.00569	0.037	0.0234	0.1	10	
Se	0.0005	< 0.0004	< 0.0004	0.0005	< 0.0002	< 0.0002	0.02	0.05	0.02
Mo	0.0003	< 0.0002	0.0004	0.0006	0.0016	0.0006	0.005	0.05	0.01
Cd	0.00002	0.00002	< 0.00002	0.00001	< 0.00001	0.00001	0.01	0.05	
Sn	< 0.0002	< 0.0002	< 0.0002	< 0.0001	0.0001	< 0.0001	2		
Ba	0.0094	0.0071	0.0027	0.0103	0.0024	0.0031	1		
Pb	0.00052	0.00032	0.00012	0.0012	0.00026	0.00091	5	20	

The results are presented in mg/L. The maximum recommended value (MRV) and maximum permissible value (MPV) are according to DL No. 236/98, while QS refers to the quality standards values established by DL No. 119/2019 (Water quality standards for reuse in irrigation to protect crops, forests, and soils).

The results indicate concentrations meaningfully lower than the maximum recommended values and below the maximum permissible values. Therefore, these parameters comply with water quality standards for irrigation. This may be due to the type of effluent treated. As it has characteristics of domestic effluent, the metal and potentially toxic loads are considerably lower than those of other types^54^. This is worth mentioning, as the characteristics of the effluent (e.g., domestic, agricultural, or industrial) can significantly impact the observed system^55^.

### Efficiency of the constructed wetland: removal efficiency

**Fig. 5:**
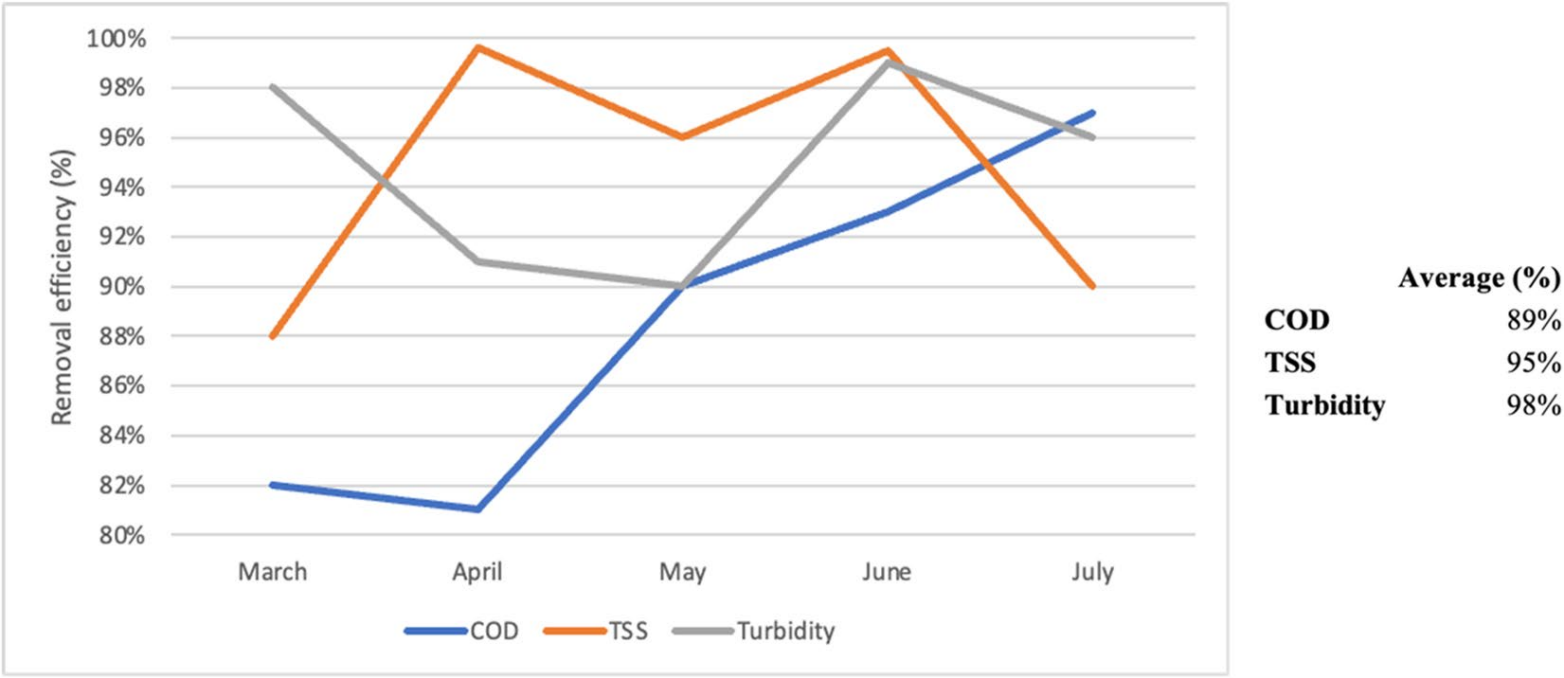
Removal efficiency (%) of chemical oxygen demand (COD), total suspended solids (TSS), and turbidity during the five campaigns (March–July).

To evaluate the CW’s performance, the removal efficiency of the previously analyzed elements was examined from the treatment entry point (P1) until its use (P3). Figure 5 shows the removal efficiency across five campaigns for three key parameters in water treatment: COD, TSS, and turbidity.

The results indicate a high removal efficiency across all evaluated parameters, particularly TSS, which achieved the highest removal efficiency of approximately 99.6% during the April campaign and the lowest at 88% in March. The average removal efficiency was 95%, aligning with the minimum efficiency requirements.

Regarding turbidity, the highest percentage was 99% during the June campaign, while the lowest was 90% in May. Similar to TSS, the average removal efficiency for this parameter was 95%.

The highest COD removal efficiency was 97% during the last campaign, while the lowest was 81% in April, with an average of 89%. It is, therefore, well above the quality standards and discharge treatment levels for sensitive environments^56^, which requires a minimum reduction of 75% in COD.

**Fig. 6:**
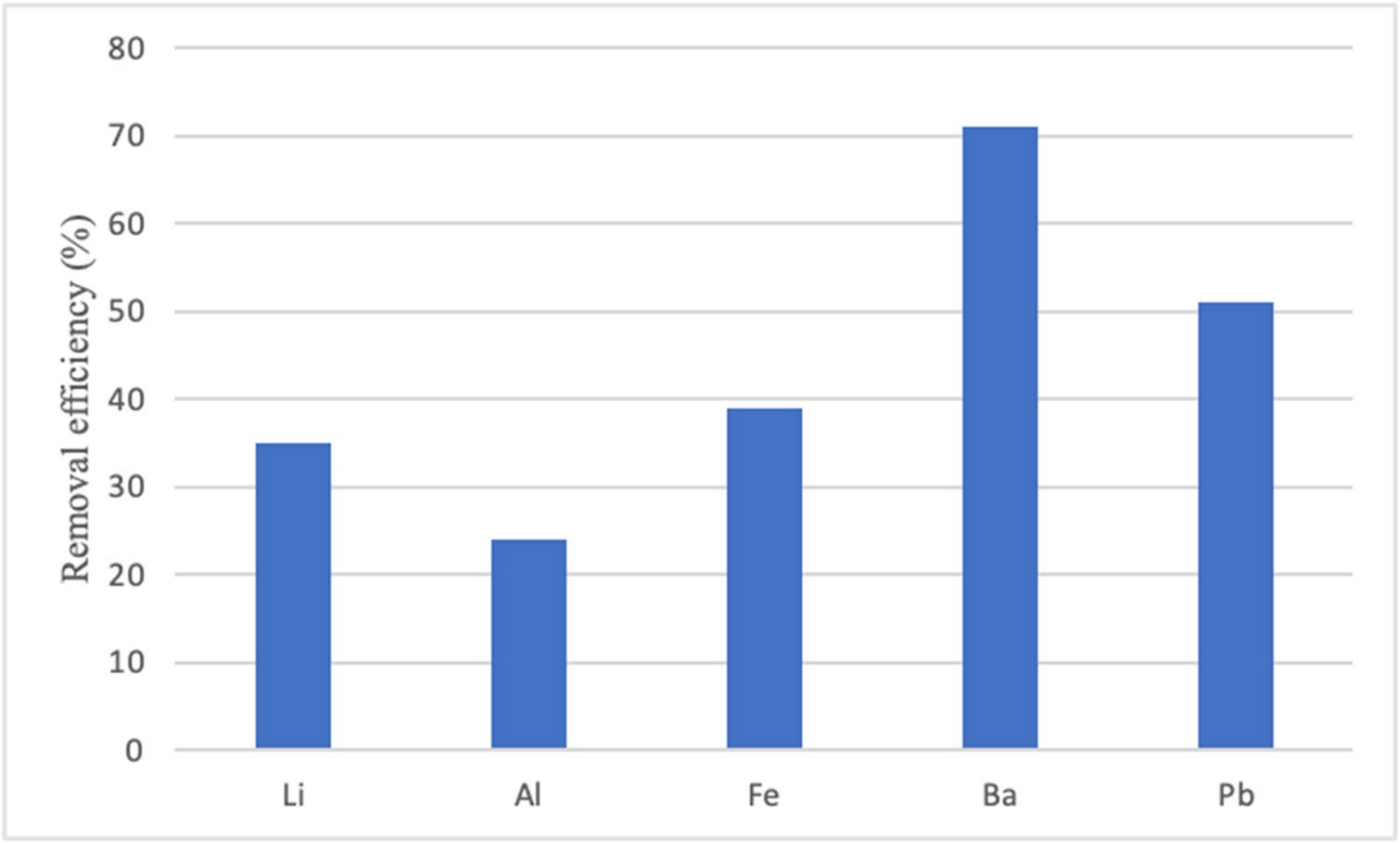
Average removal efficiency of Lithium (Li), Aluminum (Al), Iron (Fe), Barium (Ba), and Lead (Pb).

Figure 6 presents an analysis of the removal efficiency of some elements, including Li, Al, Fe, Ba, and Pb. These elements were chosen because they appear in the literature for their toxic characteristics and their potential to impact the environment and various organisms negatively^57^.

As shown, Ba and Pb have the highest removal efficiency (71% and 51%, respectively), reflecting good performance by the CW. Whether essential or not for plant nutrition, it is important to note that all these elements can also potentially become toxic at high concentrations, causing problems such as inhibiting plant growth, disrupting photosynthetic and respiratory processes, and altering enzymatic activity. This largely depends on the specific element and the plant species involved^58^. Typically, concentrations of potentially toxic elements are commonly higher in the belowground parts—roots and rhizomes—as compared to aboveground parts—stems and leaves^21^. However, when present at low concentrations, these elements can be beneficial and play a significant role in the metabolic activities of plants, contributing to processes such as growth and healthy development^59^.

### Water quality assessment

**Fig. 7:**
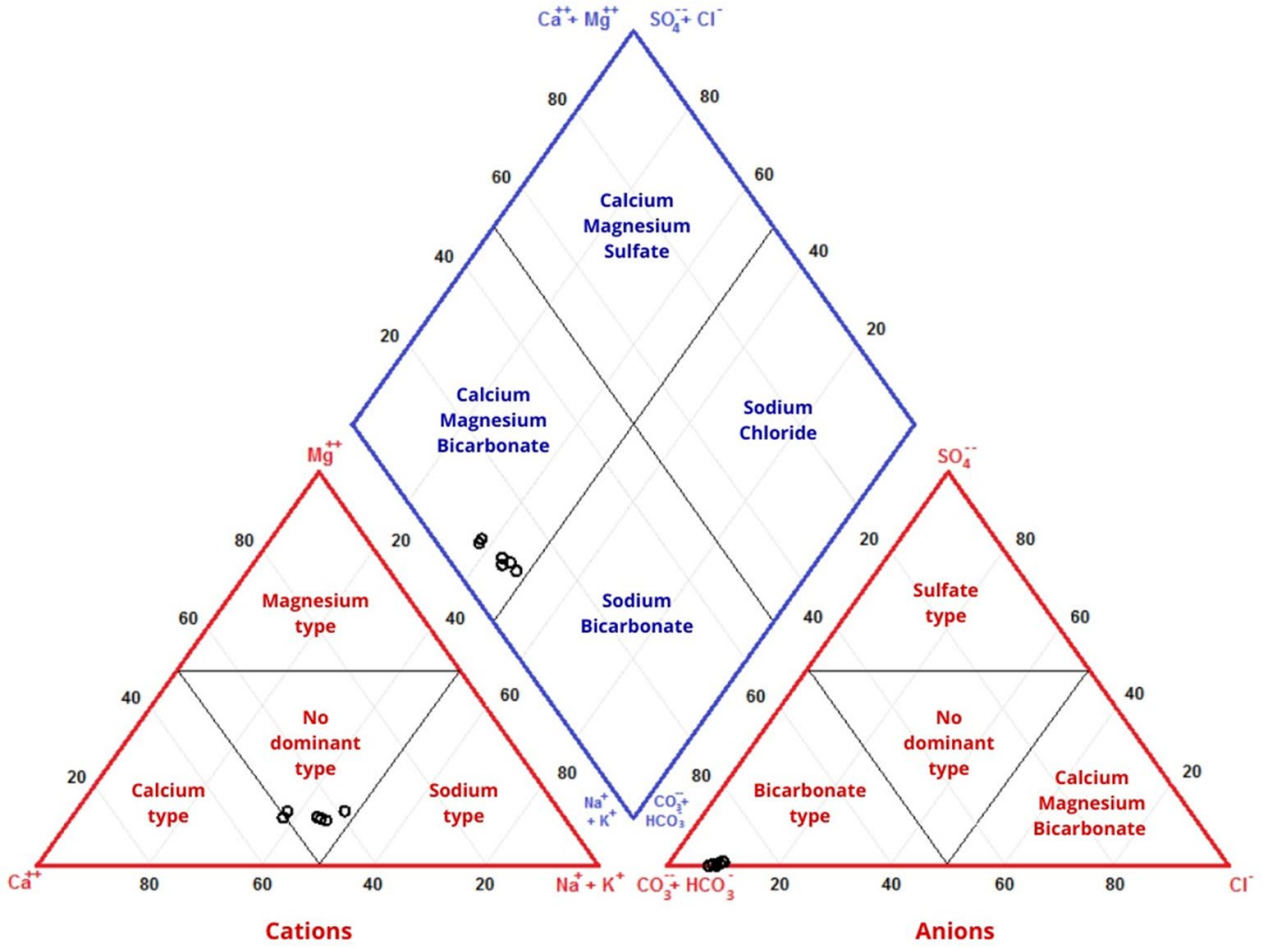
Water samples classification results—Piper diagram.

Assessing water quality in this work is essential, as it serves the study’s aim. Given the imminent threat of prolonged droughts, water reuse becomes an important factor. However, ensuring that the water meets minimum quality standards for this purpose is essential. Figure 7 displays the hydrochemical characteristics of the collected water samples. In the diagram above, water samples from the first and fourth campaigns were used, resulting in a total of six samples. Analyzing the Piper diagram indicates that most samples are categorized as mixed bicarbonate and calcium bicarbonate waters. The March samples P2 and P3, along with P1, P2, and P3 from June, fall into the mixed bicarbonate type, while sample P1 from March is classified as calcium bicarbonate water.

As observed, the highest concentration of anions is HCO_3_^−^, followed by Cl^−^, while SO_4_^−^ exhibits low concentrations. Among cations, Ca is the dominant one.

**Fig. 8:**
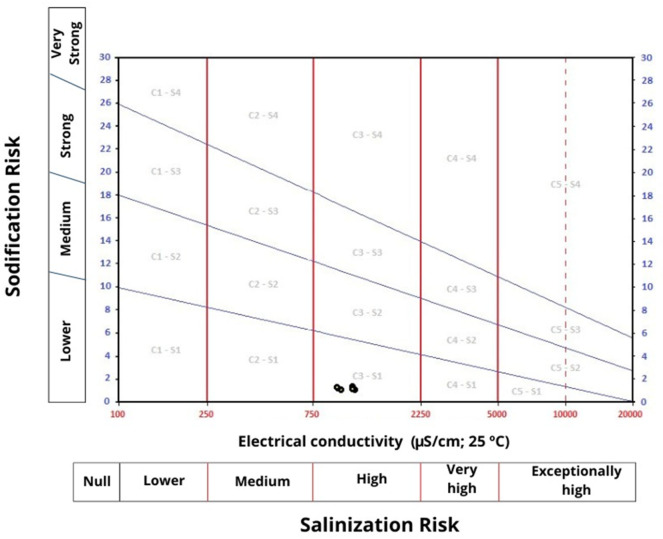
Classification diagram for irrigation water.

The risks of salinity and sodium are key factors that determine the suitability of water for irrigation^60^. Therefore, it is essential to evaluate these factors using a classic tool, where samples are analyzed based on their EC and SAR values. The classification diagram for irrigation water is illustrated in Fig. 8.

The results indicate that the samples fall within the C3-S1 group, suggesting a high risk of salinization and a low risk of sodium. This phenomenon can disrupt the properties of irrigated soil, affecting its physical and chemical characteristics and potentially leading to erosion^61^. However, the SAR value is below 2, which is under the 8 threshold set by current legislation^30^ for water quality intended for irrigation. Therefore, the water samples do not pose a sodium risk. It can thus be concluded that the sodium levels in the water samples are acceptable since the SAR is less than 2. However, due to the high risk of salinization, monitoring every six months is necessary. According to the same decree, if no events are likely to compromise water quality after two consecutive years and the analytical results remain within or below the MRV, the recommended sampling frequency may be reduced to once a year during the irrigation period.

It should be noted that the campaigns conducted during the dry season, particularly in the driest months of June and Jully, may indicate increased water evaporation in the CW and a possible decline in treatment efficiency. However, despite the reduced rainfall and higher temperatures, the removal efficiency of contaminants remained high, often exceeding levels observed during wetter periods. Although no dilution factor is involved, this phenomenon can be attributed to the concentration of the targeted elements for retention, thus enhancing the phytoremediation efficiency of the system. These results suggest that this system demonstrates sufficient resilience in regions facing significant drought challenges. Given its reliance on living treatment processes, it is adaptable and could be effectively implemented in other water-scarce areas, particularly in remote locations that require minimal maintenance.

## Conclusion

The hydrochemical properties obtained were within the expected range, considering their origin: wastewater. The analysis of the different sampling points (P1, P2, P3) demonstrated highly satisfactory performance in terms of legal compliance, particularly at point P3—the final discharge location—where nearly all the analyzed parameters fell within the Maximum Permissible Values (MPV) and Recommended Mean Values (RMV), except for total suspended solids (TSS) and ammonium (NH_4_⁺). TSS showed a single instance of non-compliance, while ammonium exceeded legal limits in all campaigns. This may be linked to the low water circulation in the system due to infrequent use, leading to extended water retention times. Additionally, inadequate maintenance of the CW system—namely, the accumulation of organic matter and the absence of thinning of the plant species *Phragmites australis*—also contributed to these outcomes.

Regarding removal efficiency and water quality, the parameters COD, TSS, and turbidity demonstrated maximum removal efficiencies of 97%, 99.6%, and 99%, respectively, surpassing the thresholds required for discharge into sensitive environments. These findings confirm the effective performance of the CW system in removing contaminants.

As for the risk of salinization, the Sodium Adsorption Ratio (SAR) remained below 2, well under the legal limit of 8, indicating a low sodium hazard. However, the results indicate a high risk of salinization, warranting semi-annual water quality monitoring.

Sampling campaigns conducted during the dry season proved to be more demanding from a hydrochemical perspective due to reduced precipitation and higher temperatures, which intensified the concentration of ecosystem-disrupting elements. Despite these challenges, contaminant removal efficiency remained high—in some cases, even exceeding the performance observed during wetter periods. These findings underscore the resilience of phytoremediation techniques, demonstrating their suitability even under conditions of water scarcity.

So, it is possible to say that the reuse of treated wastewater through constructed wetland systems presents a viable solution for irrigation purposes, contributing to sustainability goals and addressing water scarcity, particularly in regions prone to severe droughts such as the Mediterranean. Nevertheless, to ensure the long-term efficiency of the system, regular maintenance and the implementation of rigorous monitoring protocols are essential.

## Data Availability

The authors declare that the data supporting the findings of this study are available within the paper.
